# Re-Creation of Historical Chrysotile-Containing Joint Compounds

**DOI:** 10.1080/08958370802290595

**Published:** 2008-09-11

**Authors:** G. P. Brorby, P. J. Sheehan, D. W. Berman, J. F. Greene, S. E. Holm

**Affiliations:** Exponent, Inc., Oakland, California; Aeolus, Inc., Albany, California; Exponent, Inc., Spokane, Washington; Georgia-Pacific, LLC, Atlanta, Georgia

## Abstract

Chrysotile-containing joint compound was commonly used in construction of residential and commercial buildings through the mid 1970s; however, these products have not been manufactured in the United States for more than 30 years. Little is known about actual human exposures to chrysotile fibers that may have resulted from use of chrysotile-containing joint compounds, because few exposure and no health-effects studies have been conducted specifically with these products. Because limited amounts of historical joint compounds are available (and the stability or representativeness of aged products is suspect), it is currently impossible to conduct meaningful studies to better understand the nature and magnitude of potential exposures to chrysotile that may have been associated with historical use of these products. Therefore, to support specific exposure and toxicology research activities, two types of chrysotile-containing joint compounds were produced according to original formulations from the late 1960s. To the extent possible, ingredients were the same as those used originally, with many obtained from the original suppliers. The chrysotile used historically in these products was primarily Grade 7RF9 from the Philip Carey mine. Because this mine is closed, a suitable alternate was identified by comparing the sizes and mineral composition of asbestos structures in a sample of what has been represented to be historical joint compound (all of which were chrysotile) to those in samples of three currently commercially available Grade 7 chrysotile products. The re-created materials generally conformed to original product specifications (e.g. viscosity, workability, crack resistance), indicating that these materials are sufficiently representative of the original products to support research activities.

## Introduction

Joint compound was one of hundreds of products used in the United States that historically contained chrysotile (ATSDR, 2001). Chrysotile-containing joint compound was commonly used in the construction of residential and commercial buildings through the mid 1970s; however, these products have not been manufactured in the United States for more than 30 years. Little is known about the actual human exposures to chrysotile fibers that may have resulted from use of chrysotile-containing joint compounds, because few exposure and no health-effects studies specific to these products have been conducted. Historical reports from the 1970s and 1980s indicated that mixing, sanding, and sweeping of joint compound generated “substantial” quantities of dust (Rohl et al., 1975; Rhodes and Ingalls, 1976; Fischbein et al., 1979; Verma and Middleton, 1980). However, data from these studies may not accurately represent historical chrysotile exposures, for several reasons (e.g. samples were analyzed only by phase contrast microscopy (PCM), which is not capable of distinguishing chrysotile fibers from other fibers; authors frequently reported heavily loaded or overloaded filters, which can result in unreliable estimates of fiber concentrations; only short-term samples were collected, which complicates the ability to estimate long-term average exposure; and the proximity of sampling devices to dust sources may have resulted in collection of substantial fractions of large particles that are not respirable but were counted nonetheless). Because little of the original joint compound is available, and there are questions concerning whether historical samples would remain chemically stable; evaluation of the historical joint compound using current methods and technologies is not considered feasible. Thus, the small amount of joint compound that does remain cannot be used to conduct meaningful studies to better understand the nature and magnitude of the potential exposures to chrysotile that may be associated with use of these products.

Therefore, to support research activities (e.g. a biopersistence study of chrysotile-containing joint compound in rats; see Bernstein et al., 2008), two types of chrysotile-containing joint compounds historically manufactured by Georgia-Pacific LLC (GP) (or its predecessors) were produced according to the original formulations from the late 1960s. One is a joint system cement, a dry product that has to be mixed with water before using, and one is a ready-mix, which is already mixed with water and had been marketed in a ready-to-use form. The specific formulations for each of these products were chosen based on a number of considerations, including chrysotile content, availability of ingredients, and representativeness of the entire product line. Based on these criteria, a 1967 formulation for a joint system cement containing approximately 5.5% chrysotile and a 1969 formulation for a ready-mix containing approximately 4.5% chrysotile were chosen. The percentage of chrysotile in these formulations was at the upper end of the percentage chrysotile used in the entire GP product line.

## Methods

The two products were produced at Forensic Analytical Laboratories, Inc., in Hayward, California. Microscopic analysis was conducted at EMS Laboratories in Pasadena, California.

### Non-Chrysotile Materials

The ingredients used to produce the joint system cement and ready-mix are listed in Tables 1 and 2, respectively, along with the function of each ingredient in the product and the supplier of the materials used in the re-created products. These ingredients are consistent with previously published information on the composition of joint compounds (Verma and Middleton, 1980). The formula for the ready-mix is divided into two parts—one formula is for the filler, which is a mixture of dry ingredients, and one formula is for the final product, which is a mixture of the filler and wet ingredients. To the extent possible, the exact materials described in the 1967 (joint system cement) and 1969 (ready-mix) specification sheets from the same suppliers were used; however, slight modifications were necessary to accommodate changes in availability of a few of the components, material formulation, and batch size (original specifications were designed for 4,000-lb batches as compared to 10 to 20-lb batches for the re-created products).

**TABLE 1:** Joint system cement ingredients

Ingredient	Function	Mass used in reformulation (grams)	% by weight	Supplier
Limestone^a^	Filler	3640	73	Science Stuff
Mica^b^	Anti-cracking agent	800	16	Zemex Industrial Minerals
Casein	Adhesive	225	4.5	Science Laboratories; Sigma Aldrich
Borax	Alkaline source	7.5	0.15	Van Dyke Supply Co.
Soda ash	Alkaline source	20	0.4	Jacquard
Zinc oxide	Viscosity stablizer	5	0.1	Science Laboratories
Natrosol	Water retention	15	0.3	Aqualon (Hercules)
Alkanol XC (Petro AD)	Wetting agent	5	0.1	Sigma Aldrich/DuPont
Vancide 51Z^c^	Fungicide	—	—	—
Dimethyldithiocarbamate, zinc salt	Substitute for Vancide 51Z	1.875	0.038	Sigma Aldrich
Nalco 71-D5^c^	Anti-foaming agent	—	—	—
Chrysotile 7RF9^c^	Bulk	—	—	—
Chrysotile 7RF3	Substitute for 7RF9	275	5.5	Johns Manville

Note.

a 100% calcium carbonate natural chalk (CAS# 471-34-1).

b Muscovite mica.

c Ingredient substituted or eliminated – see text.

**TABLE 2:** Ready-mix ingredients

Ingredient	Function	Mass used in reformulation (grams)	% by weight	Supplier
Filler				
Limestone^a^	Filler	5255	81	Science Stuff
Mica^b^	Anti-cracking agent	845	13	Zemex Industrial Minerals
Natrosol	Water retention agent	42.25	0.65	Aqualon (Hercules)
Gelvatol 20-30 BP (Polyvinyl alcohol)	Adhesive	52	0.8	Science Laboratories; Alfa Aesar
Troysan CMP-10-Sep^c^	Fungicide	—	—	
Dowicil	Substitute for Troysan CMP-10-Sep	9.75	0.15	ET Horn Co.
Nalco 71-D5^c^	Anti-foaming agent	—	—	—
Chrysotile 7RF9^c^	Bulk	—	—	—
Chrysotile 7RF3	Substitute for 7RF9	292.5	4.5	Johns Manville
Ready-mix				
Filler	Body	5485	57	—
Elvacet 81-900 (Polyvinyl acetate emulsion)^c^	Adhesive	—	—	—
Playamul 104	Substitute for Elvacet 81-900	482	5	Forbo
Benzoflex 50	Plasticizer	31.2	0.3	Velsicol
Nalco 71-D5^c^	Anti-foaming agent	6	0.06	Nalco
Water	Solvent	3602.5	37.5	Municipal supply

Note.

a 100% calcium carbonate natural chalk (CAS# 471-34-1).

b Muscovite mica.

c Ingredient substituted or eliminated – see text.

- The original joint system cement formulation called for 0.075% Vancide 51Z (fungicide) by weight, which is no longer available in a dry formulation. Historically, Vancide 51Z was approximately a 50:50 formulation of 3n 2-mercaptobenzo-thiazole:dimethyldithiocarbamate, zinc salt. The mercaptobenzothiazole is an accelerant typically used in rubber product manufacturing, and the carbamate has fungicidal properties. A 100% formulation of the carbamate is currently available; therefore, this material was used at 0.03% by weight to produce the joint system cement (i.e. approximately half the mass called for, because the active ingredient is present at twice the concentration).
- The original joint system cement formulation called for 0.02% Nalco 71-D5 (defoamer) by weight. Nalco 71-D5 (kerosene, paraffin wax, hydrodesulfurized mineral seal oil) is a liquid ingredient and difficult to homogenize with the dry-mix materials. While the inclusion of a defoamer would be important for the 4,000-lb batches produced historically, defoaming was not an issue for the amount of material being produced as part of this effort. Therefore, Nalco 71-D5 was not included in the joint system cement.
- The original ready-mix formulation specified that Nalco 71-D5 (defoamer) be added to the dry ingredients (i.e. the filler); however, because this material is a liquid, it was added with the other wet ingredients instead.
- The original ready-mix formulation called for 0.1% Troysan CM-10-Sep (fungicide), which is no longer available. Dowicil (1-[3-chloroally]-3,5,7-triaza-1-azonia adamantine chloride) at 0.15% was used to produce the ready-mix based on fungicides used in other historical ready-mix formulations.
- The original ready-mix formulation called for Elvacet 81–900 (polyvinyl acetate emulsion), which also is no longer available. Plyamul 104, which is also a polyvinyl acetate emulsion, has the same percentage of solids as the Elvacet 81–900, and is manufactured by the company that acquired the glue manufacturing business from the original supplier of Elvacet 81–900, was used to re-create the ready-mix.

Additionally, the limestone and mica ingredients of the joint system cement and ready-mix formulations were tested for the presence of grit according to GP’s standard testing procedure for insoluble raw materials. Approximately 200 g of each material was placed on a moistened 50-mesh screen. The screen was placed under running water to allow fines to be washed through. No residue was left on the screen, indicating that grit was not present in the sample.

### Chrysotile

The 1967 and 1969 formulations for both the joint system cement and the ready-mix called for Grade 7RF9 chrysotile, which was historically obtained from the Philip Carey mine in Quebec, Canada. Because this mine is no longer operating, an alternative type of chrysotile had to be used in the re-creation of the two joint compounds. The chrysotile used was selected by characterizing the sizes and mineral types of asbestos structures in a sample of what has been represented to be the historical joint system cement, and then comparing them to the sizes and mineral types of asbestos structures in samples of three other Grade 7 chrysotile products that remain commercially available: Johns Manville (JM) 7RF3 and JM 7R05, which are from the Jeffrey Mine in Quebec, Canada, and Brazilian CB7RP. Details of this evaluation are documented elsewhere (Berman et al., in preparation); however, the methods used in the selection of an appropriate replacement fiber are summarized in the Methods section, and findings are summarized in the Results section of this paper.

Trace amounts of tremolite have been found in some samples of chrysotile from Quebec, Canada (e.g. Addison and Davies, 1990). With regard to the chrysotile used historically (Carey 7RF9), two studies have investigated the possible content of tremolite in the Carey Canadian chrysotile deposit. In 1980, Butler wrote a PhD dissertation entitled, “The Physical and Chemical Characteristics of Serpentine Rocks and Minerals.” Six samples from the Carey Mine (two ore samples and four product samples) were included in Butler’s investigation and were subjected to mineral examination by powder X-ray diffraction (XRD), optical microscopy, and electron microscopy. Tremolite was not detected in any of the samples from the Carey Mine (limit of detection not specified). In a more recent study (Gunter et al., 2007), 10 samples of ore, in-place rock, and tailings from the Carey Mine were collected and analyzed for amphiboles using powder XRD. Amphibole was not detected in nine of the ten samples (limit of detection of 100 parts per million (ppm)); amphibole was detected in the tenth sample at 500 to 1,000 ppm. Further analysis of the amphiboles in the latter sample by scanning electron microscopy (SEM) and polarized light microscopy (PLM) identified nonasbestiform amphibole consisting of approximately 50% anthophyllite and the remainder actinolite (Gunter et al., 2007).

The chrysotile used in the re-created joint compound (JM 7RF3) did not undergo a mineralogical examination similar to that conducted on the samples from the Carey Mine reported by Butler and Gunter et al. However, Williams-Jones et al. (2001) conducted a detailed investigation of the Jeffrey mine, which indicated that amphibole does not occur within the chrysotile ore itself, but instead occurs in association with intrusions (felsic dykes) within the ore (due to alteration of the serpentinite adjacent to such intrusions) and in other lithologic deposits (pyroxenite and slate) at the boundaries of the ore. The study concludes that avoiding these intrusions and bordering deposits during mining would effectively preclude the introduction of amphibole into the processed ore.

Finally, samples of the historical joint system cement and the three modern Grade 7 materials were examined by transmission electron microscopy (TEM) to characterize asbestos structures. Samples were examined according to the counting rules described in the next section, which resulted in approximately 450 structures being counted in each of three replicate samples for a total of approximately 1,350 structures per material. All of the observed structures were identified as chrysotile.

### Evaluating and Selecting Chrysotile

A sample of the historical joint system cement was homogenized and split into subsamples, and three subsamples (triple replicates) were prepared and analyzed separately. Using the Modified Elutriator Method (Berman and Kolk, 2000), dust representing the respirable fraction of particles in each sample was collected on filters that were then prepared by direct transfer for analysis by transmission electron microscopy (TEM). Asbestos structures collected on the filters were characterized using the counting rules of ISO Method 10312 (ISO, 1995) with stopping rules modified to assure counting of a sufficient number of structures (in each size category of interest) to develop representative structure size distributions from each sample, with pre-defined precision. Thus, a sufficient area of the filter was scanned while counting each size fraction of interest to assure either that a target minimum number of structures in that size fraction was observed or that the abundance of such structures waslower than a pre-established minimum.

Size distributions were characterized by estimating the fraction of total structures in each size category using the method of maximum likelihood (Cox and Hinkley, 1974), assuming that the number of structures in each category has a Poisson distribution. Details of this analysis are provided elsewhere (Berman et al., in preparation).

The size categories included in these analyses were:

- Structures shorter than 5 *μ*m
- Structures between 5 and 10 *μ*m in length that are also:
  - thinner than 0.25 *μ*m,
  - between 0.25 and 0.4 *μ* m in thickness, or
  - thicker than 0.4 *μ*m
- Structures between 10 and 40 *μ*m in length (divided into the width categories indicated above)
- Structures longer than 40 *μ*m (divided into the width categories indicated above)

This classification scheme was applied separately to each of the following categories of structure types:

- Primary structures (i.e. structures about which a clear boundary can be drawn that separates each primary structure from the others on the filter)
- Primary fibers and bundles (using the definitions of fibers and bundles in ISO 10312)
- All fibers and bundles (this includes both primary fibers and bundles, and fibers and bundles that are components of larger, more complex structures)

The overall fraction of structures that are complex (i.e. structures that are fibers and bundles in matrices or clusters) was also determined, as was the fraction of fibers and bundles contained in more complex structures.

Three samples of each of the three commercially available Grade 7 chrysotile materials were prepared and analyzed as described above for the historical joint system cement. The relative size distributions of each sample (as well as a best-estimate of the mean size distribution across each of the replicate samples of each commercial material) were then compared to size distributions similarly determined for the replicate samples of historical joint system cement, to evaluate which of the three commercially available materials was most similar to the historical material. The best-estimate size distributions (across samples) were determined using the method of maximum likelihood (Cox and Hinkley, 1974), in which the observed number of structures in each size category was assumed to have a Poisson distribution. Details of the evaluation used to define the best-combined size distributions for each sample type are provided elsewhere (Berman et al., in preparation).

### Re-Creating Joint Compounds

A sample of the chosen Grade 7 chrysotile was first homogenized and split into appropriately sized subsamples, to assure reproducibility across the re-created materials. The quantities of the other ingredients were then calculated based on the weight of the chrysotile subsample and the percentages of each ingredient specified in the original formulation. All chrysotile-containing materials were handled in a glove bag constructed with two commercially available 60 × 72-inch glove bags (Grayling EXT6072), PVC pipes, and duct tape, or in a fume hood.

### Joint System Cement

The ingredients for the joint system cement, other than the chrysotile, were weighed on appropriately sized and calibrated balances outside of the glove bag. These materials were then placed in a 5-gallon bucket with an airtight lid and stirred gently with a long, metal spoon. The bucket and lid were labeled with the product name, date, and chrysotile content. The lid was attached and the contents were mixed by rolling or turning the bucket end over end. The bucket, chrysotile, and other necessary materials (e.g. spatulas, rags) were placed into the glove bag, along with a Patterson-Kelly 1/3-cubic-foot twin-shell blender with impactor bar. The glove bag was inspected, and any tears or openings were sealed with duct tape. The chrysotile was added to the other ingredients in the bucket and the mixture was stirred gently with a spatula to incorporate the chrysotile. The material was then transferred to the twin-shell blender, and the lids were placed on the blender according to the manufacturer’s instructions. The material was blended for 10 minutes and then allowed to settle for an additional 30 to 60 seconds. The material was transferred back to the 5-gallon bucket, and the sealed bucket was removed from the glove bag and placed in a fume hood pending performance testing. Chrysotile-containing waste materials were disposed of properly, and the interior of the glove bag was cleaned using a HEPA vacuum.

### Ready-Mix

The dry ingredients for the ready-mix were weighed, mixed, and handled as described above for the joint system cement. The majority of the water and remaining wet ingredients were weighed on appropriately sized and calibrated balances and placed in the bowl of a Hobart 20-quart planetary action blender. A small amount of water was set aside as a reserve. The homogenized filler was weighed on an appropriately sized and calibrated balance, added to the top of the wet materials, and allowed to sit for 1 minute. A spatula was then used to gently mix the materials before blending for 2 to 3 minutes until the material appeared homogeneous. The remaining water was added in increments until the appropriate consistency had been achieved. The material was covered with wet paper towels and allowed to sit in the hood for 30 minutes prior to conducting performance testing.

### Product Specification Testing

After the materials were formulated, subsamples of the joint system cement and the ready-mix filler were tested according to the original product specifications; the joint system cement was mixed with water prior to testing.

#### Working Consistency

The purpose of this test was to determine the amount of water (as a percentage of dry ingredients) necessary to achieve the consistency at which the material would most likely be used. At least 30 minutes after the joint system cement was mixed with water and/or the ready-mix was prepared, a subsample was mixed with a spatula. The material should be smooth and creamy, and should slide off the spatula in one doughy lump.

#### Alkalinity

The purpose of this test was to determine the pH of the joint compound. Original product specifications provided no requirement for the pH of the ready-mix. The pH of the joint system cement should be between 8.8 and 9.2. To conduct this test, a pH electrode (Basic ph/mv/ORP, Thermo Electron Corporation, Orion 420A+) was placed in the joint compound and allowed to stabilize.

#### Working Properties

The purpose of this test was to assess the application properties of the material, including smoothness, freeness (i.e. degree to which the joint compound sticks to a knife), plasticity, and absence of grit or foreign materials. A layer of joint compound was spread about 1/16-inch thick and approximately 16 inches long on a piece of wall board using a broad knife, making several flat strokes with the knife while applying pressure. The surface of the application was inspected for lumps, specks of foreign material, or agglomerates of any of the raw materials. The surface of the material was then sheared, and the surface was observed as before. A second layer was applied and observed for freeness and plasticity. The material should be plastic, buttery, and free of grit or course particles.

#### Bonding Properties

The purpose of this test was to determine the bonding properties of the paper joint tape when using joint compound. Two 0.025-inch wide and 12-inch long feeler gauge strips (thin metal shims) were placed 3 to 4 inches apart on a piece of wall-board. A layer of joint compound was applied between the feeler gauge strips using a broad knife. A 12-inch long piece of paper tape was placed in the center of the joint compound, and the tape was bedded into the material by applying pressure strokes with the broad knife, such that excess material was squeezed out from under the tape. The material was allowed to dry for 24 hours, and two “X”s were cut through and across the tape about 3 to 4 inches from the end, using a utility knife. The utility knife was then used to peel back the edge of the tape, which was then pulled sharply. The tape should delaminate when pulled back (the product specifications for the ready-mix state that the tape should show at least 75% fiber tear when delaminated).

#### Crack Resistance

The purpose of this test was to determine the tendency of the joint compound to crack. A 3/16-inch thick and 2-inch wide stainless-steel shim was placed on the left-hand edge of a piece of wallboard. Joint compound was applied to the wallboard with a spatula, and a wedge was formed using a stiff 6-inch broad knife. The material was then placed under a forced-air fan for approximately 4 to 5 h. At most, only a few small cracks should form in the wedge of joint system cement. There should be no large fissure cracks in the wedge and no cracks in the thin section of the joint compound.

#### Color

The purpose of this test was to assess the color of the product. Joint compound was applied to a piece of wallboard and allowed to dry overnight. The joint system cement should be neutral in color, and the ready-mix should be white.

#### Viscosity

The purpose of this test was to determine the viscosity of the joint compound. This test was conducted using a Brabender Viscometer “Visco-Corder” Model VC-3 (CW Brabender Instruments, Inc., South Hackensack, NJ) adjusted to operate at 79 rpm with a 250 cm-g cartridge. Prior to testing, the Viscometer sensitivity cartridge was calibrated by the CW Brabender Instruments Company. The rotational speed was measured with an internal mechanical tachometer, which was checked periodically using a calibrated laser tachometer. The Viscometer sample cup was filled completely with joint compound, and the cup was tapped sharply on a hard, flat surface several times to remove any bubbles. The cup was placed on the Viscometer according to the manufacturer’s instructions, and the instrument was turned on. The viscosity was read after 30 seconds. The viscosity of the joint system cement was not specified in the original formula. The viscosity of the ready-mix should be 580 ± 20 Brabender Units (BU).

## Results

### Evaluation of Chrysotile Samples

Detailed findings regarding the mineral composition and size distribution of asbestos structures observed in historical joint system cement, and in the three Grade 7 chrysotile samples to which the historical material was compared, is provided elsewhere (Berman et al., in preparation). Briefly, all structures observed in the historical material and Grade 7 samples were identified as chrysotile. With regard to fiber size distribution, results indicate that, of the three Grade 7 samples, JM 7RF3 exhibits the distribution that most closely mimics that observed in samples of the historical joint system cement. The size distributions for primary fibers and bundles are presented for each of the four materials in Table 3. The historical material contains a greater proportion of long, thin fibers and bundles relative to any of the three Grade 7 samples evaluated, although the fiber characteristics of the JM 7RF3 sample appear to be similar. The longest, thinnest fibers and bundles make up substantially smaller proportions of the total distribution of fibers and bundles in either of the of the other two Grade 7 samples (i.e. JM 7R05 or Brazilian CB7RP). This is true whether the comparison is made among primary structures or total fibers and bundles (Berman et al., in preparation).

**TABLE 3:** Distribution of sizes of primary fibers and bundles in historical joint system cement and three samples of Grade 7 chrysotile

Historical joint system cement primary fibers and bundles					
	<5 *μ*m	5–10 *μ*m	10–40 *μ*m	>40 *μ*m	all lengths
<0.25 *μ*m	0.444	0.041	0.050	0.003	0.537
0.25–0.4 *μ*m	0.180	0.012	0.018	0.001	0.212
>0.4 *μ*m	0.187	0.041	0.024	0.000	0.251
all widths	0.811	0.094	0.092	0.004	1
JM 7RF3 primary fibers and bundles					
	<5 *μ*m	5–10 *μ*m	10–40 *μ*m	>40 *μ*m	all lengths
<0.25 *μ*m	0.476	0.040	0.020	0.001	0.536
0.25–0.4 *μ*m	0.162	0.004	0.005	0.001	0.172
>0.4 *μ*m	0.268	0.013	0.010	0.001	0.292
all widths	0.906	0.057	0.035	0.002	1
JM 7R05 primary fibers and bundles					
	<5 *μ*m	5–10 *μ*m	10–40 *μ*m	>40 *μ*m	all lengths
<0.25 *μ*m	0.345	0.026	0.005	0.000	0.377
0.25–0.4 *μ*m	0.125	0.008	0.002	0.000	0.135
>0.4 *μ*m	0.465	0.015	0.008	0.000	0.488
all widths	0.935	0.049	0.016	0.001	1
Brazilian CB7RP primary fibers and bundles					
	<5 *μ*m	5–10 *μ*m	10–40 *μ*m	>40 *μ*m	all lengths
<0.25 *μ*m	0.364	0.025	0.008	0.000	0.397
0.25–0.4 *μ*m	0.137	0.007	0.003	0.000	0.146
>0.4 *μ*m	0.421	0.025	0.009	0.000	0.456
all widths	0.922	0.057	0.021	0.001	1

**FIG. 1:**
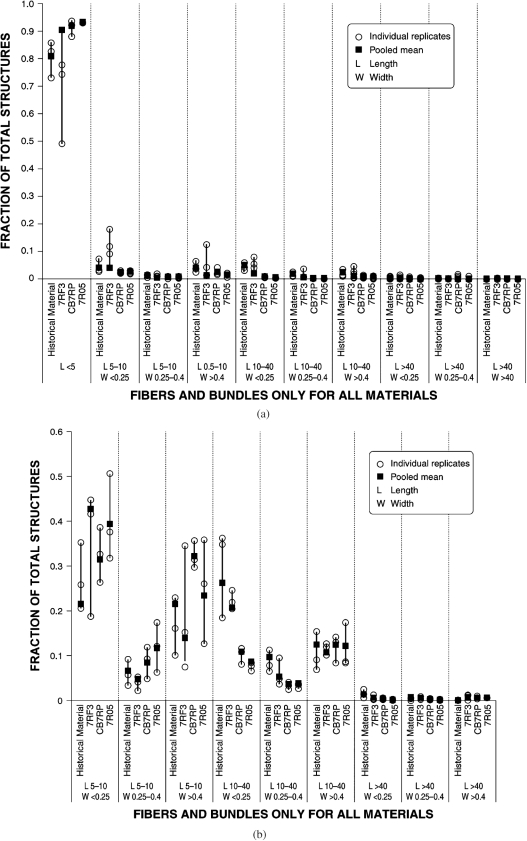
Comparision of chrysotile fiber size distributions for historical joint compound material and fiber from three commercial sources (a) all fiber lengths [above]; (b) fibers longer than 5 um [below].

An additional comparison is provided in Figures 1a and b, which augment the information in Table 3 by indicating the variation between samples of the same material (i.e. historical material or the three Grade 7 samples) and the best estimate for each material. Figure 1a includes structures of all lengths. Figure 1b excludes structures shorter than 5*μ*m, so that the relative contributions from other sizes can be better observed. Circles in these figures represent the fraction in each size category observed in each of the three individual replicates analyzed for each material. The square represents the fraction in each size category observed based on the combined (pooled) mean across the replicates for each material (determined by maximum likelihood; Berman et al., in preparation). It can be seen clearly in these figures that, especially when variation is taken into account, the proportion of each size range of structures in the historical material overlaps the proportion for every one of the corresponding size ranges in JM 7RF3, but not JM 7R05 or Brazilian CB7RP.

Overall, the distribution of fiber sizes in the JM 7RF3 sample best overlaps the size distribution observed for the chrysotile in the historical joint system cement (Berman et al., in preparation). It should be noted that the chemical characteristics of the historical material appear to interfere with preparation of the polycarbonate filters used for these analyses, such that some fibers and other particles are lost during preparation. The effect of this phenomenon, which was not observed among the Grade 7 chrysotile samples, is being evaluated further using mixed cellulose ester (MCE) filters (Berman et al., in preparation). However, this comparison was not the only criterion relied upon for selecting the chrysotile sample to be used in the re-created products. JM 7RF3 was also selected, because it clearly contains the greatest fraction of the longest fibers among the materials tested, and substantial evidence suggests that it is the longest fibers that contribute the most to asbestos-related cancer risk (Berman et al., 1995; Berman and Crump, 2003, 2008; ERG, 2003a, 2003b). Thus, using JM 7RF3 for the re-created products represents a conservative choice (i.e. tending toward maximizing potential health risks) for generating materials for future exposure and toxicity studies.

### Evaluation of Product Specification Testing

Product specifications for each formulation and the results from testing the re-created materials are listed in Table 4. The re-created joint system cement and ready-mix generally conformed to the product specifications. Specifically, the re-created joint system cement conformed to the specifications for working consistency, alkalinity, working properties, crack resistance, and color. Viscosity is not specified in the joint system cement formulation; however, the viscosity of the re-created joint system cement conforms to the specification for the ready-mix. The degree of bonding was better than current-day products but less than the product specification. The re-created ready-mix conformed to specifications for working properties, color, and viscosity, and essentially conformed to the specifications for working consistency and crack resistance. Alkalinity is not specified in the formulation, but the alkalinity of the re-created ready-mix conforms to the specification for the joint system cement. As with the joint system cement, the degree of bonding observed for the ready-mix was better than current-day products, but less than the product specification.

**TABLE 4:** Results of performance testing of re-created products

	Joint System Cement		Ready-Mix	
		
Test	Specified	Measured	Specified	Measured
Working consistency (estimated amount of water as a percentage of dry ingredients)	63−67	65	53−56	60
Alkalinity	8.8−9.2	9.1	NS	8.9
Working properties	Plastic, buttery working and free of grit or coarse particles	Smooth, plastic, buttery, no grit or particles, feathers well, sheers well	(M-971) Filler shall be very heavily bodied and possess fair plasticity. It shall be free of any coarse, gritty or undispersed particles.	Heavy-bodied with fair plasticity
			(M971/974) Compound shall be plastic, buttery working and free of any coarse, gritty or undispersed particles	Very smooth, plastic, buttery, little grit or particles, feathers well
Bonding properties	After compound has dried, tape shall delaminate when peeled back	Minimal delamination, approximately 5%−10%	Tape shall show at least 75% fiber tear when delaminated	Minimal delamination, approximately 5%−10%
Crack resistance	At most, only a couple of small cracks in the wedge	No cracks	There shall be no large fissure cracks in the wedge, and no cracks in the thin section	One deep fissure in wedge, no cracks in thin section
Color	Neutral	Off-white	White	White
Viscosity (BU)	NS	565	580 ± 20	580

*Note.* NS = not specified, BU = Brabender units.

## Discussion and Conclusions

The goal in re-creating two historical chrysotile-containing joint compounds was to generate joint compounds that were representative of the original formulations with regard to the nature of the chrysotile fibers contained in the materials and the behavior of the products during normal use. To be conservative, the percentage of chrysotile in the recreated products was at the upper end of the range of percentage chrysotile used in the entire GP product line. The majority of the ingredients used to produce the joint system cement and ready-mix were the same as those specified in the original formulations from the late 1960s, and many of these ingredients were obtained from the same suppliers. The replacement of the fungicide in both formulations, and the elimination of the anti-foaming agent from the joint system cement are not expected to have an impact on the performance of the re-created materials. Several polyvinyl acetate emulsions (i.e. glue) from multiple manufacturers were evaluated as a replacement for the originally specified material in the ready-mix. Small batches of ready-mix were formulated without chrysotile to allow for evaluation of the working properties and viscosity of the various batches. The majority of these initial batches (without chrysotile) were too wet and too thin. These batches were formulated with glues that had lower percentage solids than the glue specified in the original formulation. Once a glue with the appropriate percentage of solids was identified, the test batches exhibited improved working properties and viscosity.

Re-creating the joint system cement was straightforward, and initial test batches met the majority of the product spec-ifications. Re-creating the ready-mix was more complicated, especially with regard to the blending of the dry and wet ingredients. Small test batches, this time including chrysotile, were produced to evaluate working properties and viscosity. These initial test batches (with chrysotile) were consistently too thick, even at the upper end of the specified range of water as a percentage of dry ingredients (i.e. 53% to 56%). Subsequent test batches indicated that a ratio of approximately 60% water to dry ingredients (wt/wt) resulted in material that was of the right viscosity. One possible explanation for this difference is that the original formulation was intended for GP’s manufacturing facility in Marietta, Georgia, which has higher average humidity than our research facility in Hayward, California. Furthermore, addition of the water was best accomplished in increments rather than all at once. Approximately 90% of the water was added initially, and the remaining water was added in increments until the appropriate viscosity was achieved.

Characterization of the asbestos structures in the historical joint system cement, which is believed to contain grade 7RF9 chrysotile, and the three commercially available Grade 7 chrysotile, indicate that 1) all of the observed structures are chrysotile, and 2) the majority of the chrysotile structures in all four materials are less than 5 *μ*m in length, with relatively few structures greater than 40 *μ*m in length. The observed percentage of chrysotile structures in specific size categories varied across replicates for individual materials, although the degree of variability was larger in some cases than in others (e.g. the percentage of structures less than 5*μ* m in length ranged from approximately 50% to 80% for the JM 7RF3, as compared to approximately 92% to 93% for the Brazilian CB7RP). The percentage of chrysotile structures in any particular size category also varied across the four materials, although in most cases the ranges of values overlap. These results indicate that, of the three Grade 7 samples evaluated, JM 7RF3 is the best substitute for the 7RF9 specified in the original formulations and further suggest (at a minimum) that JM 7RF3 represents the most conservative option (in terms of health considerations) for recreating the original formulations, because it contains the greatest fraction of long structures among the available fiber products tested.

The re-created materials were tested according to the original product specifications (e.g. viscosity, workability, crack resistance). Both materials generally conformed to product specifications, indicating that the performance of the re-created products during normal use would be representative of the performance of the original material.
